# Demographic factors and retrieval of object and proper names after age 70

**DOI:** 10.1371/journal.pone.0191876

**Published:** 2018-01-25

**Authors:** Gitit Kavé, Shimon Fridkin, Liat Ayalon

**Affiliations:** 1 Department of Education and Psychology, The Open University, Raanana, Israel; 2 School of Social Work, Bar Ilan University, Ramat Gan, Israel; CONICET Cordoba, ARGENTINA

## Abstract

**Purpose:**

This research aimed to investigate whether demographic factors are similarly related to retrieval of object and proper names.

**Methods:**

The sample included 5,907 individuals above age 70 who participated in the Health and Retirement Study between 2004 and 2012. Participants were asked to name two objects as well as the US President and Vice President. Latent growth curve models examined the associations of age, education, and self-rated health with baseline levels and change trajectories in retrieval.

**Results:**

Age and education were more strongly related to retrieval of proper names than to retrieval of object names, both for baseline scores and for change trajectory. Similar effects of self-rated health emerged for both types of stimuli.

**Conclusions:**

The results show that examining object names and proper names together as indication of cognitive status in the HRS might overlook important differences between the two types of stimuli, in both baseline performance and longitudinal change.

## Introduction

A recent report shows that 30% of people above age 65 complain of difficulties in word retrieval, whereas 64% complain of difficulties in retrieval of proper names [1]. Such differential subjective reports might reflect the importance that name retrieval has in social interactions, as well as the common belief that retrieval abilities decline with age. While experimental evidence generally corroborates the subjective feeling that object names are easier to retrieve than are proper names [2], there could be several reasons for these findings. In the present study we compare baseline levels and longitudinal changes in retrieval of object and proper names in a large representative sample. We specifically examine whether retrieval of these two types of names, as well as decline in retrieval, is similarly associated with age, education, and health.

Older adults often report that proper names are more elusive than are other types of words [1, 2]. Research has shown that when people fail to retrieve object names, they tend to provide alternative words, whereas a failure to retrieve a proper name leads to a “don’t know” response [3]. The disproportionate difficulty in retrieving proper names can be explained by a difficulty in retrieving the necessary semantic information about the to-be-named individual [4]. Semantic information might help the activation of background knowledge but a name cannot be substituted by synonyms. Yet, familiarity with famous people is often cohort dependent, especially when celebrities from different fields are used, and this problem undermines some results of cross-sectional studies of proper name retrieval. This limitation can be at least partially solved in a longitudinal study in which the same participants are asked to retrieve both object names and proper names over time. In addition, this limitation can be solved by asking participants to name contemporary celebrities that are supposed to be similarly familiar to all cohorts. While previous research predicts that it would be easier to retrieve object names than proper names, studies of cognitive measures in the Health and Retirement Study (HRS) have examined these two tasks together (e.g., [5]). It is thus unclear whether the rate of decline would be similar on both tasks and whether it would be similarly affected by demographic factors. To the best of our knowledge, no analyses of HRS data have thus far separated these two measures, and we believe that it is important to do so because cognitive research suggests that aging has a differential effect on each measure.

Several demographic variables have been associated with naming. Many studies have shown that increased age is related to greater difficulties in word retrieval [6, 7]. However, longitudinal studies have mostly focused on confrontation naming of objects rather than on retrieval of proper names. For example, a study that included 541 people who were followed for up to 10 years documented a significant decrease in object naming above the age of 70 [8]. Education has been shown to affect word retrieval as well, so that individuals with higher education name more objects [9]. There is also preliminary evidence that specific health status affects naming in cognitively-intact individuals. Thus, presence of hypertension, but not diabetes, has been shown to associate with reduced naming, and presence of metabolic disease has been shown to associate with reduced action naming but not with reduced object naming [9, 10].

While education attainment is known to be related to the level of cognitive performance, there are inconsistent findings regarding the effects of education on the trajectory of cognitive decline [11–13]. Some longitudinal studies have shown that higher education is associated with slower cognitive decline, some studies have shown no association between education and the rate of decline, and yet other studies have found that the association of education with the trajectory of decline is restricted to a subgroup of participants or to specific cognitive domains [12]. Although education is related to higher performance on tests of both crystallized (e.g., verbal abilities) and fluid intelligence (e.g., working memory, speed), decline in crystallized intelligence appears to be particularly sensitive to the potential effect of education [11]. According to Lenehan et al. [13], some of the inconsistency in findings concerning the association between education and the rate of cognitive decline might relate to the statistical analyses employed, as early conclusions were based on only two time points, on analyses of variances, or on simple regressions rather than on more sophisticated analytical techniques such as latent growth curve models (LGCM).

Research has also shown a relationship between common chronic health conditions in non-demented individuals and general cognition in later life. Thus, blood pressure, diabetes, or cardiovascular disease have been associated with poorer cognitive abilities [14, 15], although the associations are mostly seen on tests that require speeded performance. In the current study, we use a measure of self-rated health. Studies suggest that such a subjective measure is superior to objective indicators of health status in predicting mortality [16, 17], because it relies on self-knowledge which might not be fully detectable by quantitative measures of health. We expect that health ratings would be associated with level of baseline performance. Nevertheless, it is unclear whether a measure of self-rated health would also predict the rate of decline. Some studies have reported no association between health and rate of decline in cognition in non-demented individuals over the age of 70 [14, 15]. Such results might reflect the fact that decline occurs at an earlier age [18] or that those who continue to participate in research are particularly healthy [19].

The aims of the current research are to compare both baseline levels and rates of decline in retrieval of object names and proper names in individuals over the age of 70. In addition, we compare the associations between age, education, and self-rated health and baseline performance as well as the associations between these demographic variables and the trajectory of change over an eight-year period. Our goal is to determine whether the same demographic factors relate to baseline and to change trajectories to the same extent for both types of stimuli. To do so, we use data from individuals who participated in five biannual waves of the HRS, and analyze these data with LGCM. We expect all demographic variables to relate to baseline performance but make no predictions as to the associations of these factors with the change trajectory or as to possible differences in their associations across the two tasks.

## Method

### Participants

The sample consisted of 5,907 individuals who participated in the Health and Retirement Study (HRS) between 2004 and 2012. The HRS is a representative panel of US citizens over the age of 50 and their spouses of any age who are tested biannually. Detailed information regarding sampling procedures have been described elsewhere [20]. The HRS is supported by the National Institute on Aging (NIA U01AG009740) and the Social Security Administration. Data collection was approved by the Institutional Review Board at the University of Michigan. The present study is based on all respondents over the age of 70, who completed the relevant tasks on at least three out of five consecutive testing waves. An additional sensitivity analysis concerns respondents who had data on all five waves conducted over the same period (n = 3,024), as well as respondents who scored at ceiling (3–4) at initial testing (n = 5,382).

Respondents were between age 70 and age 102, 86.2% of the sample were white, and 59.2% were women (for further demographic information, see Table 1). Relative to the larger sample who completed at least three waves, respondents who had data on all five waves were younger (M = 75.44, SD = 4.64), more educated (M = 12.44; SD = 3.13), and healthier according to self-report (M = 3.00, SD = 1.05).

**Table 1:** Sample characteristics and correlations between variables (n = 5,907).

	Mean (SD)	1	2	3	4	5	6	7	8	9	10	11	12
**1. Age in 2004**	77.33 (5.76)	-											
**2. Education (0–17 years)**	11.99 (3.35)	-.06**	-										
**3. Subjective health in 2004 (1–5)**	2.90 (1.09)	-.04*	.20**	-									
**4. Object names 2004**	1.90 (.31)	-.11**	.25**	.11**	-								
**5. Object names 2006**	1.87 (.35)	-.15**	.25**	.12**	.51**	-							
**6. Object names 2008**	1.87 (.36)	-.14**	.26**	.13**	.48**	.50**	-						
**7. Object names 2010**	1.86 (.36)	-.13**	.25**	.11**	.43**	.42**	.48**	-					
**8. Object names 2012**	1.87 (.37)	-.13**	.21**	.09**	.40**	.41**	.47**	.54**	-				
**9. Proper names 2004**	1.75 (.48)	-.15**	.34**	.13**	.27**	.29**	.26**	.22**	.22**	-			
**10. Proper names 2006**	1.71 (.53)	-.20**	.32**	.14**	.25**	.32**	.30**	.24**	.22**	.57**	-		
**11. Proper names 2008**	1.67 (.56)	-.23**	.30**	.14**	.20**	.23**	.32**	.26**	.24**	.50**	.54**	-	
**12. Proper names 2010**	1.41 (.62)	-.21**	.29**	.15**	.14**	.17**	.19**	.26**	.24**	.33**	.35**	.42**	-
**13. Proper names 2012**	1.49 (.62)	-.20**	.28**	.10**	.15**	.17**	.19**	.18**	.29**	.34**	.36**	.40**	.56**

Note: Maximum score on both outcome variables was 2.

*p < .05

**p < .01

### Measures

#### Object names

Two items evaluated retrieval of object names: "What do people usually use to cut paper?" (Scissors); "What do you call the kind of prickly plant that grows in the desert?" (Cactus). We used the sum of the two items as the outcome variable (range 0–2).

#### Proper names

Two items evaluated retrieval of proper names: "Who is the President of the United States right now?"; "Who is the Vice President?". We used the sum of the two items as the outcome variable (range 0–2).

#### Demographic information and health status

We analyzed the effects of age, education, and self-rated health, as assessed in 2004. Self-rated health was measured with the question: “Would you say your health is excellent, very good, good, fair, or poor?”. Responses were coded on a scale of 1 (poor) to 5 (excellent).

### Analytical approach

We first calculated descriptive statistics and correlations between variables. Next, we used Structural Equation Modeling (SEM) with Amos version 23 to estimate parallel LGCMs of the two naming outcome variables. To establish LGCMs, we followed the following steps:

#### (a) Establishing that the same constructs are measured over time

The first step prior to establishing LGCMs requires the establishment of weak factorial invariance with regard to the outcome variables. This ensures that the items are consistently related to the overall construct over time. We examined weak factorial invariance by setting factor loadings of each variable as equal across waves. Like-items were allowed to correlate across waves. The disturbances of these constructs were specified as correlated within each wave.

#### (b) Establishing the latent growth curve models

To examine the longitudinal relationships between the two outcome variables, LGCMs were fitted within the SEM framework. The model allows for the simultaneous examination of growth trends for the two outcomes of interest to examine associations between baseline (intercept) and change trajectory (slope).

This model was estimated in several steps. We first examined each outcome variable separately (running two separate LGCMs) in order to establish the change trajectories that result in the best fit to the data. We assessed models in which the change trajectory was estimated as changing linearly (e.g., the size of loadings changes at a similar amount each wave). We also assessed a non-linear model, in which the first and last waves of the data were fixed to different nonzero values and any remaining loadings were free, as well as quadratic and cubic models of change trajectories. The Akaike’s information criterion (AIC) and Bayesian information criterion (BIC) were used to compare model fit, with lower values indicating better fit.

After establishing adequate fit indices for each of the LGCMs separately, the two LGCMs were examined together as a single unconditional multivariate model. Covariances between baseline levels and change trajectories were estimated in this model.

#### (c) Examining differences between object and proper names

Once the shapes of the change trajectories were established (based on the fit indices), we examined whether the baseline (i.e., intercept) and change trajectories (i.e., slope) of the two outcome variables (i.e., object and proper names) were comparable. We used a series of models, in which (a) the baseline of the two outcome variables was restricted to be equal against an unrestricted model; and (b) the change trajectories of the two outcome variables were restricted to be equal against an unrestricted model. A significant chi-square difference indicates that the restricted model is significantly worse, and that the unrestricted model is preferable.

#### (d) Examining the role of demographic predictors

To examine the conditional model, we entered age, years of education, and subjective health ratings, as assessed in 2004, as time invariant predictors of the baseline levels and the change trajectories of the two outcome variables.

#### (e) Examining differences between object and proper names with regard to the predictors

We examined a series of restricted models to determine whether the effects of the predictors on the baseline scores and the change trajectories of the two outcome variables were comparable (for example, whether the effect of age on the baseline retrieval of object names was comparable to its effect on the baseline retrieval of proper names). A significant chi-square value indicates that the restricted model is significantly worse and that effects are different.

We followed accepted recommendations for estimation of goodness-of-fit [21]. In addition to the chi-square statistic, we report three approximate fit indices, the Tucker-Lewis Index (TLI), the Comparative Fit Index (CFI), and the Root Mean-Square Error of Approximation (RMSEA). TLI and CFI close to or above .95, combined with RMSEA of .06 or lower, indicate a reasonably good fit [22]. The significance level criterion for all statistical tests was set at .05.

#### Missing values

Missingness ranged between <1% and 15% across variables. The data were not missing completely at random. Therefore, missing values were replaced using multiple imputation (MI), which is a preferred technique for handling missing data and has numerous advantages over other approaches [23]. MI involves the generation of multiple “complete” datasets by imputing possible missing values. It then provides pooled estimates that are based on combining results across the complete datasets. After analyzing the pattern of missingness, the Markov Chain Monte Carlo (MCMC) method of the MI procedure of SPSS was applied to create 10 imputed datasets.

#### Statistical analysis

Bivariate associations were examined using pooled data from cross-tabulations, and *χ*^2^ statistics were computed using an online interactive calculation tool [24]. We conducted a multivariable analysis of the pooled data using generalized linear modeling, and specifying a main-effects-only model with robust covariance matrix estimation. Parameter estimation used a hybrid method, in which Fisher scoring iterations were performed before the use of the Newton-Raphson method, and maximum iterations were set to 100. The use of pooled data produced a similar pattern of results to that found when analyzing the original data, so only pooled data are presented here. Because our outcome variables were binary, we used optimal scaling procedures for categorical data. The idea behind optimal scaling is to assign numerical quantifications to the categories of each variable, thus allowing standard procedures to obtain a solution on the quantified variables. The optimal scale values are assigned to categories of each variable based on the optimizing criterion of the procedure in use. In an additional sensitivity analysis, we examined the same models by including only individuals who had data on all five consecutive waves (n = 3,024).

## Results

Table 1 presents the correlations between outcome and demographic variables and between outcome variables on each wave. The two outcome variables declined across the five waves and were correlated with each other across all five waves.

### (a) Establishing that the same constructs are measured over time

As a first step of our main analyses, we tested the measurement model of the two latent constructs over the five time points, with cross-wave correlations between errors of the same indicators and factor loadings of like-items constrained for equality across waves. The model demonstrated good fit to the data: χ^2^ (40, *N* = 5,907) = 908.00, *p* < .001, TLI = .918, CFI = .941, RMSEA = .061 (90%CI = .057; .064).

### (b) Establishing the latent growth curve models

Next, the unconditional model was examined. The fit indices for the various shapes of change trajectories are presented in Table 2. Importantly, the non-linear models resulted in improved fit indices. The non-linear models were thus selected to represent the trajectories of change in outcome variables in all subsequent analyses.

**Table 2:** Fit indices for all study models (n = 5,907).

Growth model	χ^2^	df	*p*	Δχ^2^	Δdf	CFI	TLI	RMSEA	90%CI	AIC	BIC
Weak factorial invariance	908.00	25	.000	NA	NA	.941	.918	.061	.057;.064	692.18	464.93
*Unconditional model (no predictors in the model)*											
*Object names*											
Linear growth specified	20.03	10	.029	NA	NA	.998	.998	.013	.004;.021	40.03	106.87
**Non-linear growth specified (fixing the loadings of the first and the last waves of data and freeing the other loadings)**	**16.87**	**7**	**.018**	**NA**	**NA**	**.998**	**.997**	**.015**	**.006;.025**	**2.87**	**129.76**
Quadratic growth specified	99.65	10	.000	NA	NA	.986	.979	.039	.032;.046	119.65	186.49
Cubic growth specified	170.07	10	.000	NA	NA	.975	.962	.052	.045;.059	190.07	256.91
*Proper names*											
Linear growth specified	708.12	10	.000	NA	NA	.897	.845	.109	.102;.116	728.12	794.96
**Non-linear growth specified (fixing the loadings of the first and the last waves of data and freeing the other loadings)**	**71.13**	**7**	**.000**	**NA**	**NA**	**.990**	**.979**	**.040**	**.032;.049**	**100.13**	**187.02**
Quadratic growth specified	255.76	10	.000	NA	NA	.964	.945	.065	.058;.071	275.76	342.60
Cubic growth specified	158.21	10	.000	NA	NA	.978	.967	.050	.043;.057	178.21	245.05
*Object names and proper names in the same unconstrained model*											
**Non-linear growth specified (object names and proper names in the same unconstrained model)**	**197.00**	**35**	**.000**	**NA**	**NA**	**.989**	**.983**	**.028**	**.024;.032**	**257.00**	**457.52**
*Differences between object names and proper names*											
Non-linear growth specified, mean baseline of object names and mean baseline of proper names are set as equal	672.22	36	.000	482.22**	1	.956	.933	.055	.051;.059	737.22	931.05
Non-linear growth specified, mean change trajectory of object names and mean change trajectory of proper names are set as equal	745.25	36	.000	548.25**	1	.951	.926	.058	.054;.061	803.25	997.08
*Age*, *education*, *and subjective health as covariates of object names and proper names*											
Non-linear growth specified, object names and proper names in the same unconstrained model and age, education, and subjective health as covariates	240.83	53	.000	NA	NA	.989	.981	.024	.021;.028	342.83	683.71
The effect of age is constrained as equal on both baseline scores	299.58	54	.000	58.76**	1	.985	.975	.028	.025;.031	399.58	733.78
The effect of age is constrained as equal on both change trajectories	269.92	54	.000	29.09**	1	.987	.978	.026	.023;.029	369.92	704.12
The effect of education is constrained as equal on both baseline scores	422.66	54	.000	181.83**	1	.978	.963	.034	.031;.037	522.66	856.86
The effect of education is constrained as equal on both change trajectories	245.91	54	.000	5.61*	1	.981	.979	.024	.021;.027	340.84	675.04
The effect of subjective health is constrained as equal on both baseline scores	244.35	54	.000	3.52	1	.989	.981	.024	.021;.028	344.35	678.54
The effect of subjective health is constrained as equal on both change trajectories	241.56	54	.000	.73	1	.989	.981	.024	.021;.027	341.56	675.76

*p < .05;

**p < .001.

Table 3 presents parameters and covariations of the final unconditional model. Higher baseline retrieval of object names was positively associated with higher baseline retrieval of proper names. A steeper change trajectory in retrieval of object names was positively associated with a steeper change trajectory in retrieval of proper names. The covariance of the baseline and change trajectory in retrieval of object names was significant, whereas the covariance of the baseline and change trajectory in retrieval of proper names was non-significant. These results suggest that those who retrieve more object names at baseline decline less steeply than those who retrieve fewer object names at baseline, but no such a relationship between a person’s baseline level and change trajectory in retrieval of proper names was found.

**Table 3:** Parameters and covariations of the final unconditional model (n = 5,907).

Parameter	Mean (SE)	Variance (SE)
Object names: Baseline	1.90 (.004)*	.05 (.002)*
Object names: Change trajectory	-.02 (.001)*	.00 (.0003)*
Proper names: Baseline	1.75 (.006)*	.15 (.004)*
Proper names: Change trajectory	-.09 (.003)*	.01 (.0005)*
Covariance	Covariance estimate	95% CI
Baseline-Baseline	.043*	.01;.07
Change trajectory-Change trajectory	.002*	-.001;.005
Object names: Baseline-Change trajectory	.002*	-.002;.004
Proper names: Baseline-Change trajectory	-.002	-.005;.001

*p < .001

### (c) Examining differences between object and proper names

Baseline scores of object names were higher than were baseline scores of proper names (see the chi-square differences in Table 2). In addition, a comparison of the two change trajectories showed that scores of object names were less likely to decline than were scores of proper names.

### (d) Examining the role of demographic predictors

Age, education, and subjective health ratings at baseline were entered as potential predictors of the baseline and change trajectories of the two outcome variables. Table 4 presents standardized regression weights predicting baseline and change trajectoreis of object and proper names based on the final conditional model. As can be seen in the table, younger age was associated with a higher baseline score for both outcome variables. Higher levels of education and better subjective health ratings were also associated with higher baseline scores on both tasks. In addition, younger age, higher levels of education, and better subjective health ratings were associated with more moderate change trajectories for both outcome variables.

**Table 4:** Standardized regression weights predicting baseline scores and change trajectories of object names and proper names based on the final conditional model (n = 5,907).

	Β	95% CI
*Object names*: *Baseline*		
Age	-.12**	-.16;.-.09
Education in years	.31**	.27;.34
Subjective health (1–5)	.10**	.06;.13
*Proper names*: *Baseline*		
Age	-.19**	-.23;-.16
Education in years	.40**	.37;.43
Subjective health (1–5)	.10**	.07;.13
*Object names*: *Change trajectory*		
Age	-.31**	-.35;-.28
Education in years	.11**	.07;.14
Subjective health (1-)	.09*	.05;.12
*Proper names*: *Change trajectory*		
Age	-.34**	-.38;-.31
Education in years	.07*	.03;.10
Subjective health (1–5)	.08*	.04;.11

*p < .01

**p < .001

### (e) Examining differences between object and proper names with regard to the predictors

A series of constrained models was performed to evaluate whether the associations of age, education, and subjective health ratings with the baseline and change trajecotry differed for the two outcome variables (see the chi-square difference tests between the unrestricted and restricted models in Table 2). The association of age with the baseline level of object names was weaker than was its association with the baseline level of proper names. The association of education with the baseline level of object names was weaker than was its association with the baseline level of proper names. The associations of subjective health ratings with baseline scores were similar across the two tasks. In addition, the asscociation between age and change trajectory was weaker for object names than it was for proper names, and the same was true for education. In contrast, the association of subjective health ratings with the change trajectories of both outcome variables was not significantly different.

### Sensitivity analyses

In an additional sensitivity analysis, we restricted the analysis to those who had complete data on all five testing waves. Results were similar, with very few exceptions: age was not a significant predictor of the baseline score of object names, and education was not a significant predictor of the change trajectory of object names. The second sensitivity analysis of individuals who retrieved 3 or 4 names at initial testing led to very similar results as the ones reported for respondents who had data on at least three waves. Detailed results are available upon request.

## Discussion

Previous analyses of cognitive measures on the HRS examined object names and proper names together, implicitly assuming that their similarities were greater than their differences [5]. Our findings show that retrieval of object names was easier than was retrieval of proper names, with higher baseline scores on the object naming task. In addition, the trajectory of change was more moderate for object names than it was for proper names. These results corroborate earlier findings of a disproportionate difficulty in retrieving proper names in old age [2].

Cognitive studies that compared retrieval of object names and names of famous people suggested that naming of people is harder mostly because the recognition of famous people from pictures activates less background knowledge [4]. However, the current study involved no pictures. It is possible that the difference between naming these two types of stimuli lies in the fact that object names have a rich semantic network, which is not available to proper names. Indeed, the definition used to elicit the names of objects in the HRS provided some semantic cues, unlike the request for proper names, which involved no context. This difference in cuing reflects the way in which names of objects and people are represented in our mental lexicon, since proper names are stored as unique and arbitrary entities with no semantic support [25, 26]. In addition, object names are acquired earlier in life than are most proper names. Indeed, processing of objects and faces of famous people are subject to the effect of age-of-acquisition [27], and could be aided by the cumulative frequency of retrieval over one’s life that strengthens representations. Thus, while the participants of the current study knew the names of scissors and cactus throughout their lives, the names of the President and Vice President were learned later in life. Note, however, that the trajectory of change in retrieval was best described with a non-linear model for both types of stimuli rather than for proper names alone. This finding suggests that familiarity or lack of familiarity with elected figures cannot fully explain the pattern of results.

Despite the differences between the cognitive demands of the two types of retrieval, our analyses of the demographic variables showed that production of both object and proper names was related to the same factors. Hence, age, education, and self-rated health were associated with baseline scores as well as with change trajectories on both tasks. The data show that younger age, higher education, and higher self-reported health associated with better baseline scores as well as with more moderate decline over time, whether participants were asked to name objects or to retrieve the names of the President and Vice President. These findings are in line with previous studies in which older adults were shown to retrieve fewer object names as well as fewer proper names than did younger adults [2]. The results are also in line with reports that education is related to naming performance [9], and that individuals with higher education decline less steeply than are individuals with lower education on at least some cognitive tasks [12]. Moreover, our results replicate previous findings that demonstrated associations between health status and naming scores [10].

Yet, while the same demographic variables were related to retrieval of object names as well as proper names, their associations differed across stimuli type. Specifically, age and education were less strongly associated with baseline retrieval of object names than with baseline retrieval of proper names, and the same was true with regard to their association with the trajectory of decline. We speculate that older and less educated adults followed the news less closely than did younger and more educated people. Because knowledge of object names is well established and does not depend on current political interest, the effects of age and education were weaker for retrieval of object names than for retrieval of proper names. In contrast, self-rated health was similarly associated with baseline performance on the two tasks, as well as with the trajectory of change on the two tasks. While self-rated health is a good predictor of mortality [17], it could be too general to incur a differential effect on each of the naming tasks studied here. Although changes in naming in healthy older adults might be related to risk factors for brain damage [9, 10], there is no reason to assume that these risk factors lead to specific lesions that cause difficulties in one type of stimuli but not the other.

Furthermore, the baseline level of naming was associated with the change in performance on the object naming task, but no equivalent association was found for retrieval of proper names. That is, individuals who started high on the object naming task declined more mildly on this task than did individuals who started with lower scores on this task. No similar associations were found for the retrieval of proper names. These findings might reflect the protective effects of cognitive reserve [28, 29], with greater reserve leading to higher baseline levels and mitigating the rate of decline. It is possible that cognitive reserve is more important for retrieval of object names than it is for retrieval of proper names, because object names are acquired earlier in life and do not change over time.

Importantly, while the two tasks differed in baseline scores, in change trajectories, and in their associations with demographic factors, participants who retrieved more object names also retrieved more proper names, and this positive correlation was seen at baseline as well as across all testing waves. In addition, a steeper decline in one outcome variable was associated with a steeper decline in the other variable. These findings suggest that the two tasks measure related retrieval skills. According to the Transmission Deficit Hypothesis [2, 30, 31], aging weakens the connections between a word's semantic representation and its phonological representation. A weak connection transmits too little excitation and thus a given representation cannot reach the threshold necessary for activation, resulting in a production failure. This theory predicts that older adults will find it difficult to retrieve words across a variety of tasks, whether retrieval is tested for objects or for proper names.

We acknowledge that the analyses presented here have some limitations. First, naming was tested with only few items, so that possible decline had a restricted range. This limitation is a byproduct of the size of the sample and is offset by the strengths of a longitudinal study that included thousands of observations. Second, any longitudinal study involves a test-retest practice effect. It is possible that the practice effect was stronger for object names than for proper names because the names of objects did not change over the eight-year follow up, while the names of the President and Vice President did. Third, the HRS involves a short survey rather than an in-depth cognitive investigation, limiting inferences about the mechanisms that underlie retrieval of object and proper names. However, we believe that the analysis of demographic factors highlights important differences between retrieval of the two types of stimuli that cannot be studied in a small-scale cross-sectional study. Finally, the fact that participants had to retrieve different proper names after the 2008 elections, while object names did not change at that point is a limitation of our study. The names of the President and Vice President were chosen because they were supposed to be equally familiar to all participants, yet future research should use names that are not only equally familiar at a given time, but that remain stable and involve no new learning.

In conclusion, the results of the current study further demonstrate the disproportionate difficulty associated with retrieval of proper names in healthy old age. They also show that examining object names and proper names together as indication of general cognitive decline or of a general decline in naming ability might overlook important differences between the two types of stimuli. As retrieval of the two types of stimuli is impaired in dementia [1, 32], future research should investigate whether one type of task is more helpful in clinical diagnosis of individuals with different demographic characteristics.
